# Social Determinants of Health and Behavioral Risk Factors in the Tuberculosis-Type 2 Diabetes Syndemic: A Narrative Review

**DOI:** 10.7759/cureus.116346

**Published:** 2026-09-16

**Authors:** Archana Banda, Kim Pedrigal, Savannah Jayne, Jyotsna Chawla

**Affiliations:** 1 Osteopathic Medicine, Nova Southeastern University Dr. Kiran C. Patel College of Osteopathic Medicine, Clearwater, USA; 2 Foundational Sciences, Nova Southeastern University Dr. Kiran C. Patel College of Osteopathic Medicine, Clearwater, USA

**Keywords:** bidirectional screening, chronic diseases, global health, lifestyle interventions, preventive measures, social determinants of health (sdoh), syndemic, tuberculosis, type 2 diabetes mellitus

## Abstract

The co-occurrence of tuberculosis (TB) and type 2 diabetes mellitus (T2DM) represents a growing global syndemic that disproportionately affects populations with limited access to healthcare and preventive resources. T2DM is an established independent risk factor for TB, while social determinants of health (SDoH) further contribute to disease susceptibility, delayed diagnosis, and poor clinical outcomes. This narrative literature review examines the impact of SDoH and modifiable lifestyle factors on the TB-T2DM syndemic and highlights opportunities for intervention. A literature review was conducted using PubMed and Google Scholar with keywords including “Type 2 Diabetes,” “Tuberculosis,” “Social Determinants of Health,” “health literacy,” “access to healthcare,” “smoking,” and “diet.” Existing literature demonstrates that health literacy, socioeconomic status, nutrition, and healthcare accessibility are major contributors to the dual disease burden. Smoking has been associated with impaired beta-cell function, worsened glycemic control, and increased susceptibility to TB infection and recurrence. In contrast, Mediterranean dietary patterns, structured exercise, and lifestyle modification have been shown to improve glycemic control and reduce systemic inflammation. Populations from lower socioeconomic backgrounds remain particularly vulnerable because of compounding barriers, including malnutrition, overcrowding, and limited healthcare access. Addressing behavioral risk factors and SDoH through early detection, patient education, and public health interventions is essential to mitigating the TB-T2DM syndemic. An interdisciplinary approach integrating clinical care, preventive medicine, and global health policy is critical to improving long-term outcomes in affected populations.

## Introduction and background

Type 2 diabetes is a chronic condition affecting approximately 38 million Americans [1]. The etiology involves a complex interplay of genetic, dietary, and lifestyle factors [2]. While genetics and ethnicity are non-modifiable risk factors, modifiable risk factors such as lifestyle management should be targeted to reduce the risk of complications. The disease may remain asymptomatic or undiagnosed for prolonged periods, complicating prompt diagnosis, particularly among underserved populations with limited healthcare access. Clinicians play a pivotal role in motivating patients to proactively reduce their risk, given the significant cardiovascular, microvascular, immune, and other systemic ramifications of this metabolic condition. Key behavioral interventions include weight management, dietary modification, sleep optimization, and smoking cessation. Patient education should emphasize early detection of symptoms and strategies to maintain optimal glycemic control, including the use of continuous glucose monitoring devices, digital health apps, and routine finger-stick glucose monitoring. 

*Mycobacterium tuberculosis* is the bacterium that causes tuberculosis (TB), a disease that primarily affects the lungs and spreads through airborne respiratory droplets [3,4]. Once infected, individuals can have latent TB, active TB, or drug-resistant TB [3]. Individuals may be asymptomatic or present with symptoms including prolonged cough, weakness, weight loss, or fever [3,4]. Diagnostic testing for TB includes Mantoux and purified protein derivative (PPD) skin tests, interferon-gamma release assays (IGRAs), tuberculosis-specific antigen skin tests (TBSTs), and molecular nucleic acid amplification tests (NAATs). However, each diagnostic modality possesses inherent limitations. There is also the Bacillus Calmette-Guérin (BCG) vaccine for TB. WHO-recommended since 1974, this is a live-attenuated vaccine effective in preventing miliary TB and meningitis, especially when given to infants and children [3]. TB is generally curable with prompt diagnosis and appropriate multidrug therapy: rifampin, isoniazid, pyrazinamide, and ethambutol [3]. Around the world, an estimated 10.6 million people are living with TB in areas including Southeast Asia, Africa, and the Western Pacific [3]. In the US, the Centers for Disease Control reported a 16% increase in TB cases from 2022 to 2023 [3]. 

T2DM was the most reported medical risk factor among people with active TB disease [5]. This interplay is further complicated for patients with both conditions due to a higher likelihood of treatment failure [6]. Consequently, this syndemic highlights the importance of addressing risk factors for T2DM early before disease progression, which increases immune system compromise and susceptibility to tuberculosis infection. TB is a curable disease and T2DM is a preventable chronic disease. Both diseases have shown increasing prevalence in the US and globally in recent years, often affecting overlapping vulnerable populations, making this syndemic a significant global public health concern. It is of utmost importance for clinicians to ensure both conditions are detected promptly to reduce complications and improve health outcomes. Bi-directional screening involves automatically screening for one disease when a patient tests positive for the other, i.e., a patient has a hemoglobin A1C of 9.0 and is therefore screened with a PPD for TB. Based on current research, we hypothesize that early diagnosis through appropriate bi-directional screening and effective management of T2DM through targeted lifestyle interventions can significantly disrupt the TB-T2DM syndemic. This review utilizes an interdisciplinary approach to elucidate the role of behavioral dimensions, social determinants of health (SDoH), and international collaborations in decreasing the prevalence of this syndemic. 

## Review

Methods

This narrative review aims to gain a clearer understanding of the type 2 diabetes mellitus and tuberculosis syndemic, with an emphasis on modifiable shared behavioral dimensions, public health strategies, and global community efforts. This was conducted using PubMed and Google Scholar with keywords including “Type 2 Diabetes Mellitus,” “Tuberculosis,” “Social Determinants of Health,” “health literacy,” “access to healthcare,” “smoking,” and “diet.” Relevant articles from the past 10 years were included. However, some articles included in this review were published earlier; they were retained because they contained foundational clinical information that remained relevant to the scope of this review. Articles selected included the keywords above and were related to T2DM, TB, or both.

Behavioral dimensions of tuberculosis (TB) and type 2 diabetes mellitus (T2DM)

The Impact of Lifestyle Modifications (Weight Loss, Diet, Sleep, and Smoking Cessation)

Based on the myriad of complications patients with T2DM face, especially the adverse outcomes when they develop TB, the prevention of this non-communicable disease needs to be prioritized, with lifestyle modification being the most effective primary prevention method in the US. T2DM remains a substantial global public health burden, demanding increased attention to modifiable lifestyle risk factors, notably diet quality and quantity, weight, sedentary lifestyle, stress, and poor-quality sleep [7,8]. The importance of lifestyle modification in T2DM management should not be underestimated given its potential to regulate the immune system, notably keeping tuberculosis in its latent phase. 

For instance, weight loss is an evidence-supported method of improving the outcomes of type 2 diabetes, specifically weight loss of at least 7% of the original weight, which is the most effective [9,10]. By managing obesity, it has been shown to delay the progression of patients with pre-diabetes being diagnosed with type 2 diabetes [9]. For patients interested in beginning their weight-loss journey and demonstrating readiness for change, it is critical to approach these conversations in a non-judgmental and empathetic manner to promote rapport and longitudinal results. Additionally, clinicians must inquire about any socioeconomic barriers to care that patients face, as well as their current understanding of the complications of long-standing obesity spanning across various body systems. Shared decision-making also enables clinicians to work alongside patients as they develop strategic, measurable goals encompassing various aspects of weight loss, including nutrition, eating habits, level of physical activity, behavioral counseling, medications, and surgical procedures [9]. 

Given the pivotal role of weight reduction in mitigating the risk of T2DM, bariatric surgery is indicated in specific clinical scenarios for individuals with severe obesity when conservative lifestyle interventions fail to result in sustained clinically significant weight loss. Patients with T2DM and a BMI over 30 are considered surgically eligible candidates for bariatric surgery. Clinical studies have shown its potential to induce remission of T2DM in 33-90% of individuals and improve control [11,12]. However, bariatric surgery has associated risks such as nutritional deficiencies, along with setbacks, notably the costs and risk of detrimental complications [13]. Overall, sustainable lifestyle interventions remain essential for long-term glycemic control and weight management; it is essential to adopt a targeted approach to lifestyle changes.

Existing evidence suggests that adopting a balanced Mediterranean diet, along with carbohydrate restriction, may help prevent T2DM over the long term [12,14]. The diet involves daily consumption of vegetables, whole-grain bread, and fish and nuts as the main source of protein [15]. This specific diet has been shown to reduce central obesity while providing anti-inflammatory and antioxidant compounds, a benefit that would boost immunity against TB [15]. In an analysis of over 100,000 participants, adhering to a Mediterranean diet resulted in a 20 % risk reduction for T2DM [15]. In contrast, red meat, processed meat, and sugar-sweetened drinks increase the risk of T2DM [16]. By improving their diet, T2DM patients can decrease their risk of serious infections such as TB.

The metabolic consequences of inadequate sleep are frequently underestimated. However, it has a cascade of repercussions on one's holistic health. A disrupted circadian rhythm predisposes patients to increased cortisol levels, reduced insulin resistance, disruption of the appetite-stimulating hormone ghrelin and the satiety hormone leptin, and an increased risk of obesity [17]. The metabolic consequences of inadequate sleep are frequently underestimated for metabolic function and serve as a modifiable risk factor for the prevention of metabolic diseases [18]. 

Smoking cessation represents another important preventive strategy for T2DM, which impairs beta-cell function, thereby impairing glycemic regulation [19]. Given the macrovascular and microvascular complications associated with tobacco use, healthcare professionals should strongly promote smoking cessation interventions to decrease mortality and improve the overall prognosis of T2DM [20]. Smoking not only worsens chronic diabetic complications but also has been shown to increase the risk of pre-diabetes and diabetes [21]. Both diabetes and smoking are risk factors for TB, with active and passive smoking also being associated with an increased risk of TB [22,23]. Smoking cessation can therefore be used as a strategy to help alleviate unwanted adverse effects in patients who are currently diagnosed with either T2DM or TB. It can also be used as a preventive strategy to ensure that patients who are diagnosed with either TB or T2DM do not become further immunocompromised and increase their risk of acquiring another disease. As clinicians, the importance of smoking cessation in order to mitigate the risk of relapsing TB and also control T2DM is thus all the more relevant.

Social determinants of health 

Although behavioral dimensions play an important role in TB and T2DM, it is equally important to examine the impact of social determinants of health (SDoH), which are factors outside conventional genetics that impact health. These determinants include healthcare accessibility, occupational environment, income, access to food, health literacy, and living environment, which are determined by the environment patients are born into or live in [24]. Due to the airborne transmission of tuberculosis, addressing the underlying social and environmental influences remains essential. The most notable factors are poor quality of life, poor housing, overcrowding, population explosion, undernutrition, smoking, alcohol abuse, lack of education, large families, and lack of awareness regarding the causes and transmission of TB [25]. 

Social determinants of health affect not only tuberculosis outcomes but also influence glycemic control in type 2 diabetes [26]. Notably, T2DM serves as an independent risk factor for tuberculosis. Existing literature supports the numerous associations between nutritional status, crowding, aging, economics, smoking, pollution, and alcohol and the occurrence of TB infection [27]. Out of these factors, the primary ones that will be explored are socioeconomic status, education, healthcare access, nutrition, and lifestyle. Individuals from lower socioeconomic backgrounds tend to be more vulnerable to tuberculosis and T2DM due to the compounding effects of decreased health literacy, limited access to healthcare, and malnutrition. Health providers must educate and empower T2DM patients to take control of their health since active TB can induce a state of hyperglycemia, which diminishes glucose tolerance and insulin resistance [28]. Another modifiable aspect that needs to be emphasized with patients is switching to a balanced diet composed of lower glycemic index foods to decrease one’s likelihood of developing T2DM, which may, in turn, decrease the chance of developing TB. As mentioned previously, T2DM is associated with impaired immune function, making individuals more susceptible to developing a TB infection. Exposure to ambient air pollution further complicates this issue since it is associated with an increased risk of drug-resistant tuberculosis [29]. As shown, the bi-directional relationship between T2DM and TB makes it critical to proactively address the converging social determinants of health.

Public health strategies of tuberculosis (TB) and type 2 diabetes mellitus (T2DM) in a global context

In addition to the behavioral and social determinants of health explored, it is critical to investigate the continued need for public and global health initiatives centered on mitigating and managing the rise of this syndemic. The interlink of personal protective measures, social determinants of health, and global community efforts for both conditions is illustrated in Figure 1*. *

**Figure 1:**
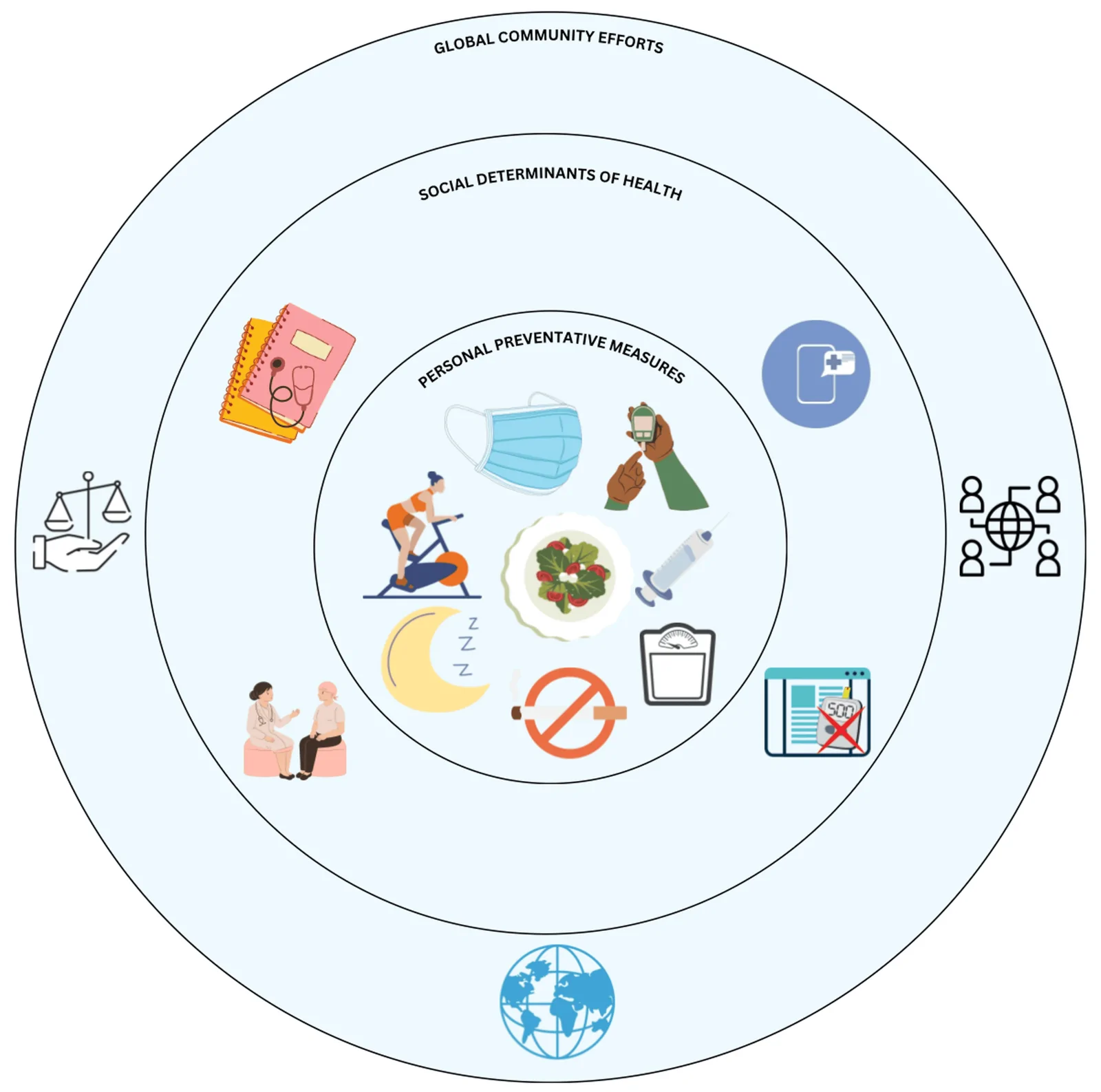
Overview of the interlink between personal preventive measures, social determinants of health, and global community efforts for TB and T2DM. Prevention of TB and T2DM involves routine healthy lifestyle practices (balanced diet and exercise), vaccination, and utilizing personal protective equipment (PPE). SDoH, especially health literacy and access to care, are key factors. Global community efforts consist of collaborative frameworks that address TB and T2DM on an international scale. This includes documents co-signed by different countries, projects headed by international unions, and guidelines to better address these intertwined conditions as a growing global burden. The illustration was created with BioRender.com. T2DM: type 2 diabetes mellitus, TB: tuberculosis.

Public Health Strategies 

As the prevalence of T2DM continues to rise globally, it is pertinent to address the proactive public health measures that can be implemented to promote healthier communities. The primary strategies for T2DM prevention are centered on early detection, education, and increased advocacy, as shown in Figure 1 [30].

Mobile health applications are increasingly being utilized as tools to prevent systemic complications stemming from uncontrolled T2DM. These technologies can improve patient engagement by presenting medical information in an accessible format. By enhancing health literacy, they empower patients to implement preventive measures, ultimately helping to decrease the global burden of this chronic condition. Also, these apps allow for a streamlined approach to various lifestyle management strategies, including pharmacotherapy, diet, blood glucose monitoring, and exercise. Even though it remains critical to develop management plans in person with health providers, there are often barriers, such as decreased access to health care and transportation to the clinic [31]. Consequently, mobile health technologies may enhance diabetes self-management [32]. Utilizing mobile apps can also be beneficial since it allows patients to increase pharmacotherapy compliance by creating reminders for their medications while logging symptoms. Additionally, patients can share real-time data, such as their blood sugar levels, diet, and physical activity, with their trusted physicians, allowing for digital communication and enhanced clinical decision-making. Despite challenges from rapidly evolving digital technologies, existing literature shows that these technology interventions empower patients to optimize their health and increase engagement and long-term well-being [32]. By giving patients, the tools to manage their chronic condition themselves, these interventions also allow them to take a hands-on approach to preventing serious complications, such as TB.

Early and effective management of T2DM is essential given the myriad of complications, including diabetic neuropathy impacting the somatic and autonomic peripheral nervous systems [33]. Optimized glycemic control results in a decreased risk of uncontrolled systemic complications. Despite the role genetics has in the pathogenesis of type 2 diabetes, patients must be empowered and educated on the controllable risk factors. Since health literacy is a social determinant of health, physicians must personalize clinical recommendations to their patients’ level of understanding, allowing for optimized adherence and outcomes [34,35]. The role of education in T2DM management cannot be understated, especially in disadvantaged social groups experiencing additional barriers to healthcare. Cross-sectional studies have shown that participants with adequate health literacy were able to recognize symptoms of T2DM accurately, whereas participants with inadequate health literacy had an increased rate of chronic conditions [35]. As shown, health literacy levels remain one of the most effective methods of delaying diabetes-related development of complications [30].

Aside from valuable insights derived from mobile apps, it is pertinent to address the role that quality health education plays in both tuberculosis and type 2 diabetes. Often, patients in underdeveloped or rural areas lack access to simplified medical knowledge, which serves as a barrier to acting in managing their health and preventing the development of diseases. Emerging artificial intelligence technologies may further enhance care; providers can use this technology to develop personalized treatment algorithms and predictive models that identify at-risk patient populations [36].

Global Community Efforts 

The association between TB and T2DM was first observed about 100 years ago, and then “rediscovered” in the 1990s [37]. This interaction between communicable and non-communicable diseases that affect similar populations makes them a double burden [38,39]. TB has been shown to occur largely in lower/middle-income and resource-poor countries such as India, Pakistan, and the Democratic Republic of the Congo [40]. The incidence of T2DM is also increasing in lower-income regions, and the age-standardized disability adjusted life year (DALY) rates are highest in regions such as Latin America, Sub-Saharan Africa, and Southeast Asia [41]. As such, the probability of the incidence rate of co-morbid TB and T2DM is increasing in Sub-Saharan Africa and South Asia. Coordinated international efforts are required to address the continuing interconnection between these two diseases, whether it be through more international and regional studies, bi-directional screenings, or global health initiatives. 

In 2011, the World Health Organization and the International Union Against Tuberculosis and Lung Disease (aka The Union) came together to make a document centered on the global burden of TB and T2DM. The Collaborative Framework for the Care and Control of Tuberculosis and Diabetes (2011) was a document aimed to “assist policymakers, public health practitioners and clinicians in understanding how to decrease the joint burden of diabetes and TB” [42]. The framework emphasized strengthening collaboration between existing TB and diabetes programs, since both diseases are so intertwined. It also acknowledged the limitations in the studies and data currently available, intending to update the framework in 2015. The recommendation in management strategies emphasizes early detection and screening tests, with an integrative coordination between the healthcare staff tasked with the care of patients with TB and T2DM [42]. The global burden of these two diseases is unevenly distributed, requiring varying levels of implementation or adaptation of the proposed guidelines based on existing systems in each region. The document also references and builds on previous collaboration initiatives/programs used for a joint tuberculosis-type 2 diabetes mellitus initiative that proved to be effective [42]

In 2015, the Indonesian Ministry of Health with the Union, and the World Diabetes Foundation came together at a summit to sign the Bali Declaration [37]. The Bali Declaration was a global initiative, a declaration signed by a hundred global health professionals with a clear stance on the danger and threat this co-epidemic has on “progress against tuberculosis” and the necessity to act now [43]. The declaration emphasized expanded bi-directional screening, making treatments for both diseases affordable, a movement to eliminate the stigma surrounding TB and T2DM, and more [44]. This screening method has shown a link between TB and T2DM, notably among people with TB, with the overall prevalence being 16.2% [45]. These findings emphasize the ongoing need for further exploration in bi-directional screening to identify concurrent disease states, emphasizing the potential for age-specific T2DM screening methods.

On a broader spectrum, in 2015, the United Nations’ 17 Sustainable Development Goals (SDGs) were established in “The 2030 Agenda for Sustainable Development” [46]. The goal of the document was to provide a “shared blueprint for peace and prosperity for people and the planet, now and into the future” [46]. Goal 3 of the SDGs is to “Ensure healthy lives and promote well-being for all at all ages” with specific targets/indicators-Target 3.3 being “By 2030, end the epidemics of AIDS, tuberculosis, malaria, and neglected tropical diseases and combat hepatitis, water-borne diseases, and other communicable diseases” and Target 3.4 “By 2030, reduce by one-third premature mortality from non-communicable diseases through prevention and treatment and promote mental health and well-being” [46]. Tuberculosis is mentioned as an indicator of Target 3.3, and diabetes mellitus is mentioned as an indicator of Target 3.4 [47]. 

In 2013, the European Union (EU) addressed the growing concern for the TB-T2DM syndemic by funding the TANDEM project [48]. This consortium was dedicated to two central hypotheses: (1) improvement in screening and management of T2DM in TB patients will impact control of comorbidity, and (2) hyperglycemia in T2DM impacts TB susceptibility and outcome [37,49]. TANDEM has field sites in four TB-endemic locations with rapidly rising rates of T2DM--Romania, Peru, South Africa, and Indonesia, combined with laboratories in four European countries--Germany, the United Kingdom, the Netherlands, and Romania [49]. TANDEM's goal of collecting data to elucidate and fill the gap in the literature on the pathophysiology of TB and T2DM can hopefully be used to inform policy and guidelines for the TB-DM syndemic. The TANDEM project began in February 2013 and was closed in July 2017 [49]. International efforts recognizing and addressing the growing co-occurrence of these two diseases are highlighted in the various projects and initiatives endorsed by the World Health Organization, the Union, the United Nations, the European Union, the Indonesian Ministry of Health, and the World Diabetes Foundation, as shown in Figure 2. 

**Figure 2:**
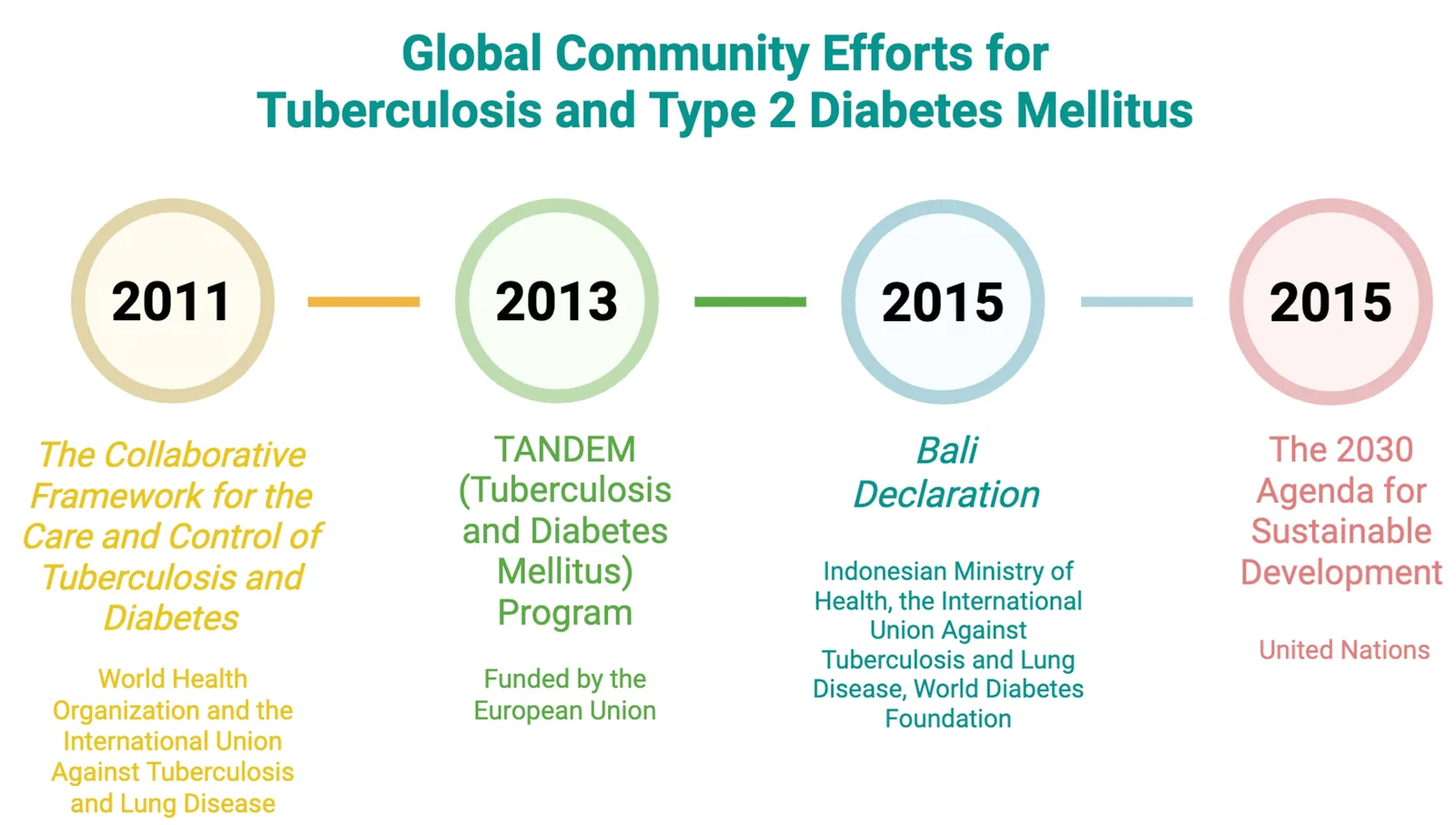
Overview of the global community efforts for tuberculosis (TB) and type 2 diabetes mellitus (T2DM). This figure shows the various global community efforts that were enacted from 2011 to 2015 with the intention of addressing the global syndemic between type 2 diabetes mellitus and tuberculosis. In 2011, the World Health Organization and the International Union Against Tuberculosis and Lung Disease created the Collaborative Framework for the Care and Control of Tuberculosis and Diabetes. In 2013, TANDEM was created by the European Union. In 2015, the Bali Declaration was created by the Indonesian Ministry of Health in collaboration with the International Union Against Tuberculosis and Lung Disease and the World Diabetes Foundation. Also in 2015, the United Nations set into motion the 2030 Agenda for Sustainable Development. The illustration was created with BioRender.com. TANDEM: Tuberculosis and Diabetes Mellitus Program, TB: tuberculosis, T2DM: type 2 diabetes mellitus.

Discussion

Given the above findings, the importance of bi-directional screening in T2DM and TB patients is critical to reducing and preventing morbidity. Lifestyle modifications can have a positive impact on the prevention of T2DM by losing weight, eating a balanced diet, sleeping an adequate amount, etc. T2DM patients can reduce their risk of long-term disease complications and help boost their immunity against infections such as TB. Social Determinants of Health play an important role not only in the development of T2DM but also in TB. By addressing and empowering patients to improve their various modifiable risk factors along with social drivers of health, clinicians can impact both disease states. Finally, T2DM and TB are both diseases of importance on the international stage, with international collaboration more focused research can be elucidated on the most at risk populations, as shown in Figure 2.

Limitations

The limitations of this review should be considered when interpreting the findings. First, the literature search relied on a predefined set of keywords, including "Social Determinants of Health," which may have excluded studies analyzing relevant social determinants of health using different terminology. Consequently, some contributing pathways or emerging evidence may not have been captured. Second, due to the evolving research centered on this syndemic, additional mechanisms and therapeutic targets may have been identified after the completion of the literature search. Third, only English-language publications were included, introducing the potential for language bias and limiting the inclusion of relevant studies published in other languages. Also, as a narrative review, there may have been selection bias in literature identification. Given the focus on TB-T2DM prevention, the existing differences in healthcare systems and epidemiological patterns across settings may limit the generalizability of the findings. Finally, this review did not include a formal statistical meta-analysis or quantitative assessment of study quality and risk of bias. 

## Conclusions

The co-occurrence of tuberculosis (TB) and type 2 diabetes mellitus (T2DM) represents a complex syndemic that disproportionately impacts populations with limited access to healthcare and preventive resources. While genetics influence an individual's susceptibility to T2DM, modifiable behavioral dimensions such as weight loss, dietary practices, sleep hygiene, and smoking cessation remain critical targets for reducing disease burden and improving outcomes. Social determinants of health, including healthcare accessibility, food security, and socioeconomic status, further exacerbate existing disparities present in this syndemic. Based on this analysis of the behavioral and social dimensions of this syndemic, it is critical for healthcare professionals to implement bi-directional screening by testing diabetic patients with TB symptoms for TB and screening all TB patients for diabetes at treatment initiation. Clinicians should prioritize patient education and health literacy initiatives, especially for patients at risk of developing both conditions through education about early detection, bi-directional risks, and the compounded complications of this syndemic. Effectively addressing the TB-T2DM syndemic consequently requires coordinated action through more collaborative international and regional efforts, bi-directional screening programs, and comprehensive global health initiatives.
